# Relative efficacy, safety and immunogenicity of bivalent versus monovalent COVID-19 mRNA booster vaccines: a randomized trial

**DOI:** 10.21203/rs.3.rs-10935317/v1

**Published:** 2026-09-14

**Authors:** Sufia Dadabhai, Taraz Samandari, Bo Zhang, Aaron Hudson, Nonhlanhla N. Mkhize, Asa Tapley, Jessica Andriesen, Penny L. Moore, Zvavahera Chirenje, Peter James Elyanu, Joseph Makhema, Ethel Kamuti, Harriet Nuwagaba-Biribonwoha, Jia Jin Kee, Jiani Hu, Katekani Baloyi-Oseh, Parth Shah, Kentse Khuto, Maurine D. Miner, Richard Lessells, Sinethemba Bhebhe, Tandile Hermanus, Haajira Kaldine, Azwidihwi Takalani, Margaret Yacovone, Laura Polakowski, John Hural, Leigh H. Fisher, Craig A. Margaret, Florence Aweyo, Sharlaa Badal-Faesen, William L. Brumskine, Soritha Coetzer, Rodney Dawson, Sinead Delany-Moretlwe, Katherine M. Gill, Mina C. Hosseinipour, Steve Innes, Priya Kassim, Emmanuel George Kafulafula, Fatima Laher, Nelly M. Mandona, Moelo Malahleha, Vongane L. Malulike, Matsontso Mathebula, Grace Mboya, Essack Mitha, Kathryn Mngadi, Pamela Mda, Tumelo Moloantoa, Nivashnee Naicker, Vimla Naicker, Annet Nanvubya, Maphoshane Nchabeleng, Walter Otieno, Elsje L. Potgieter, Disebo Potloane, Zelda Punt, Jamil A. Said, Yashna Singh, Mohammed S. Tayob, Deo O. Wabwire, Myron S. Cohen, M. Juliana McElrath, Erica Andersen-Nissen, Guido Ferrari, James G. Kublin, Linda-Gail Bekker, Peter B. Gilbert, Nyaradzo M. Mgodi, Philip L. Kotze, Yunda Huang, Nigel Garrett, Glenda E. Gray, Lawrence Corey

**Affiliations:** Johns Hopkins Research Project, Johns Hopkins Bloomberg School of Public Health, Blantyre, Malawi; Department of Microbial Pathogenesis & Immunology, College of Medicine, Texas A&M, College Station, USA; Vaccine and Infectious Disease Division, Fred Hutchinson Cancer Center, Seattle, USA; Vaccine and Infectious Disease Division, Fred Hutchinson Cancer Center, Seattle, USA; SA MRC Antibody Immunity Research Unit, School of Pathology, Faculty of Health Sciences, University of the Witwatersrand, Johannesburg, South Africa; National Institute for Communicable Diseases of the National Health Laboratory Service, Johannesburg, South Africa; Department of Medicine, University of Washington, Seattle, USA; The Desmond Tutu HIV Centre, University of Cape Town, South Africa; Vaccine and Infectious Disease Division, Fred Hutchinson Cancer Center, Seattle, USA; Vaccine and Infectious Disease Division, Fred Hutchinson Cancer Center, Seattle, USA; SA MRC Antibody Immunity Research Unit, School of Pathology, Faculty of Health Sciences, University of the Witwatersrand, Johannesburg, South Africa; National Institute for Communicable Diseases of the National Health Laboratory Service, Johannesburg, South Africa; Wits Infectious Diseases and Oncology Research Institute, Faculty of Health Sciences, University of the Witwatersrand, Johannesburg, South Africa; Centre for the AIDS Programme of Research in South Africa, University of Kwa-Zulu Natal, Durban, South Africa; Clinical Trials Research Centre, University of Zimbabwe, Harare, Zimbabwe; Baylor College of Medicine Children’s Foundation-Uganda, Kampala, Uganda; Botswana Harvard AIDS Institute, Gaborone, Botswana; Centre for Infectious Disease Research in Zambia (CIDRZ), Lusaka, Zambia; ICAP at Columbia University, Eswatini Prevention Center, Mbabane, Eswatini; Department of Epidemiology, Mailman School of Public Health, Columbia University, New York, USA; Vaccine and Infectious Disease Division, Fred Hutchinson Cancer Center, Seattle, USA; Vaccine and Infectious Disease Division, Fred Hutchinson Cancer Center, Seattle, USA; Hutchinson Centre Research Institute of South Africa, South Africa; Public Health Sciences Division, Fred Hutchinson Cancer Center, Seattle, USA; Hutchinson Centre Research Institute of South Africa, South Africa; Vaccine and Infectious Disease Division, Fred Hutchinson Cancer Center, Seattle, USA; KwaZulu-Natal Research Innovation & Sequencing Platform, School of Laboratory Medicine and Medical Sciences, University of KwaZulu-Natal, Durban, South Africa; SA MRC Antibody Immunity Research Unit, School of Pathology, Faculty of Health Sciences, University of the Witwatersrand, Johannesburg, South Africa; National Institute for Communicable Diseases of the National Health Laboratory Service, Johannesburg, South Africa; SA MRC Antibody Immunity Research Unit, School of Pathology, Faculty of Health Sciences, University of the Witwatersrand, Johannesburg, South Africa; National Institute for Communicable Diseases of the National Health Laboratory Service, Johannesburg, South Africa; SA MRC Antibody Immunity Research Unit, School of Pathology, Faculty of Health Sciences, University of the Witwatersrand, Johannesburg, South Africa; National Institute for Communicable Diseases of the National Health Laboratory Service, Johannesburg, South Africa; Hutchinson Center Research Institute of South Africa, Chris Hani Baragwanath Academic Hospital, Soweto, South Africa; National Institute of Allergy and Infectious Diseases, National Institutes of Health, Bethesda, USA; National Institute of Allergy and Infectious Diseases, National Institutes of Health, Bethesda, USA; Vaccine and Infectious Disease Division, Fred Hutchinson Cancer Center, Seattle, USA; Vaccine and Infectious Disease Division, Fred Hutchinson Cancer Center, Seattle, USA; Vaccine and Infectious Disease Division, Fred Hutchinson Cancer Center, Seattle, USA; Kisumu CRS Clinical Research Center, Kenya; Clinical HIV Research Unit, Faculty of Health Sciences, University of Witwatersrand, South Africa; The Aurum Institute NPC, Rustenburg CRS, South Africa; Vanderbilt University, Department of Medicine, Nashville, TN, USA; Synexus CRS, Helderberg, South Africa; University of Cape Town Lung Institute Clinical Research Site, South Africa; Wits RHI, University of the Witwatersrand, Johannesburg, South Africa; The Desmond Tutu HIV Centre, University of Cape Town, South Africa; University of North Carolina at Chapel Hill School of Medicine and UNC Project Malawi, Malawi; The Desmond Tutu HIV Centre, University of Cape Town, South Africa; Perinatal HIV Research Unit Soweto Kliptown, South Africa; Johns Hopkins Research Project-Kamuzu University of Health Sciences, Blantyre, Malawi; Perinatal HIV Research Unit, Faculty of Health Sciences, University of the Witwatersrand, South Africa; University of North Carolina, Global Projects Zambia (UNC GPZ), Zambia; Synergy Biomed Research Institute, South Africa; MERCLINCO, Middelburg, South Africa; MeCRU (Medunsa Clinical Trial Site), South Africa; Kisumu CRS Clinical Research Center, Kenya; Newtown Clinical Research, Johannesburg, South Africa; The Aurum Institute, South Africa; Nelson Mandela Academic Clinical Research Unit CRS, Walter Sisulu University, South Africa; Wits Health Consortium, PHRU-Matlosana, South Africa; CAPRISA eThekwini CRS, Durban, South Africa; Tongaat CRS, South Africa Medical Research Council, South Africa; UVRI-IAVI HIV Vaccine Program LTD. CRS, Uganda; MeCRU, Sefako Makgatho Health Sciences University/NHLS, South Africa; US Army Research Directorate-Africa, Kisumu, Kenya; Synexus Stanza Clinical Research Centre (CRS), South Africa; CAPRISA Vulindlela CRS, Durban, South Africa; PHOENIX Pharma (Pty) Ltd, South Africa; Moi University Clinical Research Center (MUCRC), Kenya; The Desmond Tutu HIV Centre, Institute of Infectious Diseases and Molecular Medicine, University of Cape Town, South Africa; MERCLINCO, South Africa; MU-JHU Research Collaboration CRS, Kampala, Uganda; University of North Carolina, Chapel Hill, USA; Vaccine and Infectious Disease Division, Fred Hutchinson Cancer Center, Seattle, USA; Cape Town HVTN Immunology Laboratory, Hutchinson Centre Research Institute of South Africa, Cape Town, South Africa; Duke University Human Vaccine Institute, Duke University School of Medicine, Durham, NC, USA; Center for HIV/AIDS Vaccine Immunology, Duke University School of Medicine, Durham, NC, USA; Vaccine and Infectious Disease Division, Fred Hutchinson Cancer Center, Seattle, USA; The Desmond Tutu HIV Centre, University of Cape Town, South Africa; Vaccine and Infectious Disease Division, Fred Hutchinson Cancer Center, Seattle, USA; Clinical Trials Research Centre, University of Zimbabwe, Harare, Zimbabwe; Qhakaza Mbokodo Research Clinic, Ladysmith, South Africa; Vaccine and Infectious Disease Division, Fred Hutchinson Cancer Center, Seattle, USA; The Desmond Tutu HIV Centre, University of Cape Town, South Africa; Centre for the AIDS Programme of Research in South Africa (CAPRISA), University of KwaZulu–Natal, Durban, South Africa; Discipline of Public Health Medicine, School of Nursing and Public Health, University of KwaZulu-Natal, Durban, South Africa; South African Medical Research Council, Cape Town, South Africa; Perinatal HIV Research Unit, Faculty of Health Sciences, University of the Witwatersrand, Infectious Diseases and Oncology Research Institute, South Africa; Vaccine and Infectious Disease Division, Fred Hutchinson Cancer Center; Departments of Medicine and Laboratory Medicine & Pathology, University of Washington, Seattle, USA

## Abstract

The relative efficacy, safety, and immunogenicity of monovalent mRNA-1273 (ancestral WA1) versus bivalent mRNA-1273.222 (ancestral plus omicron BA.4/BA.5) boosting have not been studied prospectively where HIV and SARS-CoV-2 seroprevalence are high. CoVPN 3008 (NCT05168813) was a randomized, double-blinded trial in seven African countries during omicron waves, enrolling people with HIV (PWH) or comorbidities associated with severe COVID-19 who had previously received mRNA-1273. Participants were randomized 1:1 to intramuscular monovalent or bivalent booster and routinely PCR-tested for SARS-CoV-2. Between 10/2022 and 03/2023, 3,988 participants (3,086 PWH) received bivalent (n=2012) or monovalent (n=1976) booster. By month 12, 293 COVID-19 cases and 221 asymptomatic infections occurred, including one severe case. COVID-19 rates did not differ between arms (hazard ratio [HR] 0.92, 95% CI 0.73–1.15, P=0.45). Combined COVID-19 and asymptomatic infection was 15% lower with the bivalent (239 vs. 275; HR 0.85, 0.71–1.01, P=0.07), and asymptomatic infection alone was 24% lower (HR 0.76, 0.59–1.00, P=0.05). Neutralizing antibody titers to BA.4/5 and XBB.1.5, measured in 100 PWH per arm, boosted higher in bivalent recipients (P=0.026 and 0.002). Adverse events were similar. In predominantly PWH, the bivalent booster was well tolerated, elicited superior immunogenicity against omicron variants, and appeared to protect against asymptomatic infection but not COVID-19.

## INTRODUCTION

mRNA vaccines have been pivotal in the response to the COVID-19 pandemic. Monovalent vaccines targeting the ancestral WA1 SARS-CoV-2 strain were deployed worldwide after trials demonstrated >94% efficacy against symptomatic disease, and up to 100% efficacy in preventing severe disease and death after two doses three to four weeks apart^1,2^. However, as with all COVID-19 vaccine platforms, waning immunity and escape from prior immunity by new SARS-CoV-2 variants – particularly omicron and its sub-lineages – reduced vaccine efficacy, especially in the elderly and the immunocompromised^3,4^.

Following the global dominance of the omicron strain, international regulatory bodies approved bivalent mRNA vaccines directed against the BA.1 and/or BA.4/5 strains in 2022 to be used as boosters after the primary series^5,6^. They were designed to induce broader immunity, and while their efficacy was subsequently established in the general population, they were not rigorously studied in immunocompromised persons, with available evidence limited to observational designs or vaccinations in different calendar periods^7,8^, creating a key evidence gap.

Globally, 41 million people live with HIV (PWH), mostly in East and Southern Africa. PWH face increased risk for severe COVID-19, including hospitalization and death, even after adjusting for other factors^9,10^. Prior clinical trials of COVID-19 vaccines included few PWH, and no large trials have evaluated boosters in this group.

We conducted a randomized, double-blinded clinical trial (RCT) to determine the safety and relative efficacy of a bivalent versus monovalent mRNA boost in a large cohort of PWH in Africa. Given documented concerns about immunocompromised persons with asymptomatic infection transmitting SARS-CoV-2, and the potential for mRNA vaccines to reduce asymptomatic acquisition, we examined efficacy against both asymptomatic infections and symptomatic COVID-19^11,12^. Given the role of neutralizing antibodies as a correlate of protection against COVID-19^13^, we assessed immune responses to circulating omicron sub-lineages in a subset of volunteers.

## RESULTS

**Figure 1.**
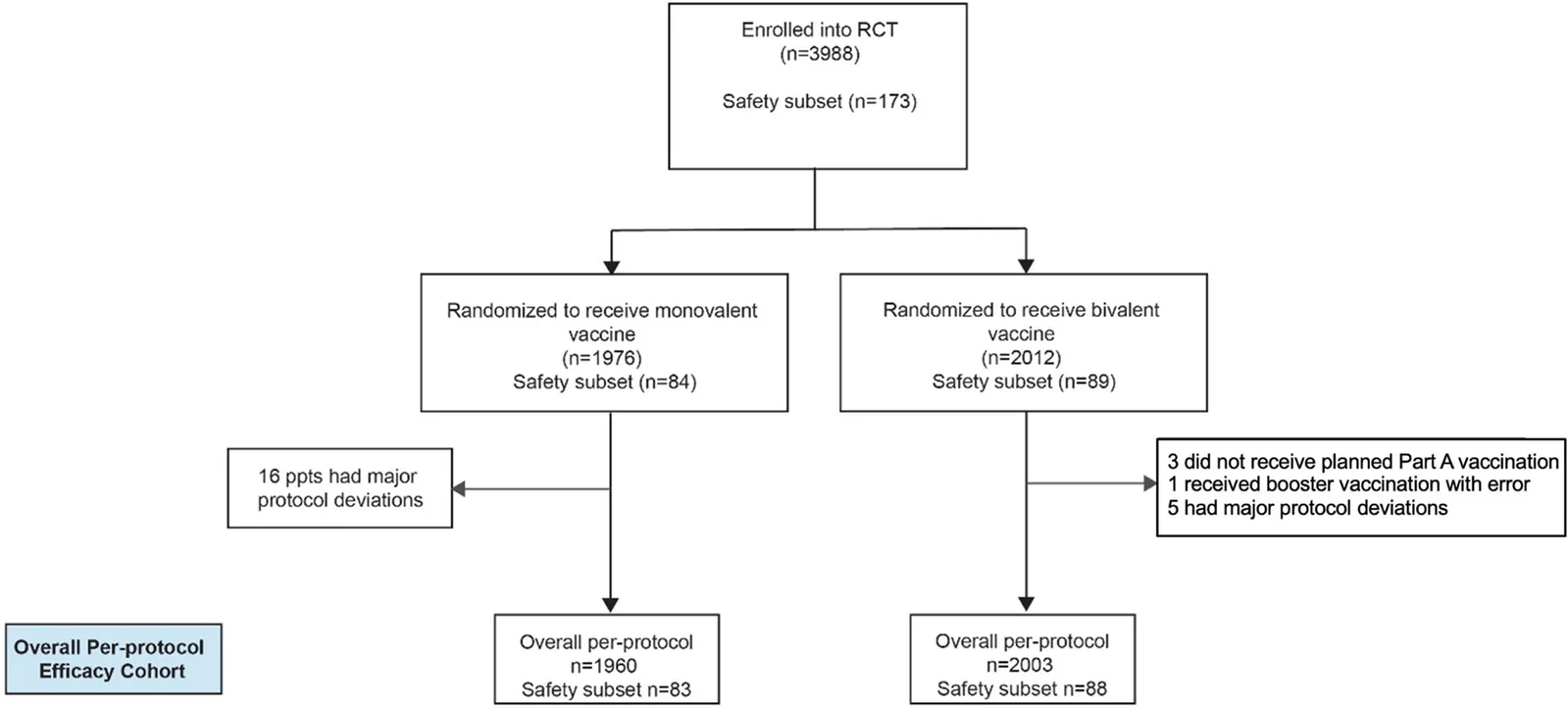
Trial Profile (Trial Participants Flow Diagram)

Between 24 October 2022 and 17 March 2023, 3,988 participants were enrolled and received a COVID-19 booster vaccination under the randomization scheme: 1,976 in the monovalent arm and 2,012 in the bivalent arm (Fig. 1). Of these, 3,086 (77.4%) were PWH and 902 (22.6%) were PWoH. Overall, 67.9% were born female; median age was 37 years (range 18–80); 3,491 (87.5%) had hybrid immunity and 497 (12.5%) had vaccine-induced immunity; 2,884 were from South Africa (72.3%) while 1,104 (27.7%) were from Botswana, Malawi, Kenya, Uganda, Eswatini or Zambia; and 11.9% had a history of TB (Table 1). Among PWH, 96.1% were receiving ART, median CD4 count was 653 cells/mm3 (IQR 454, 887), 5.5% had a CD4 count <200 cells/mm3, and 18% had HIV viremia (≥50 copies/ml). Participants’ baseline characteristics were balanced by arm.

**Figure 2.**
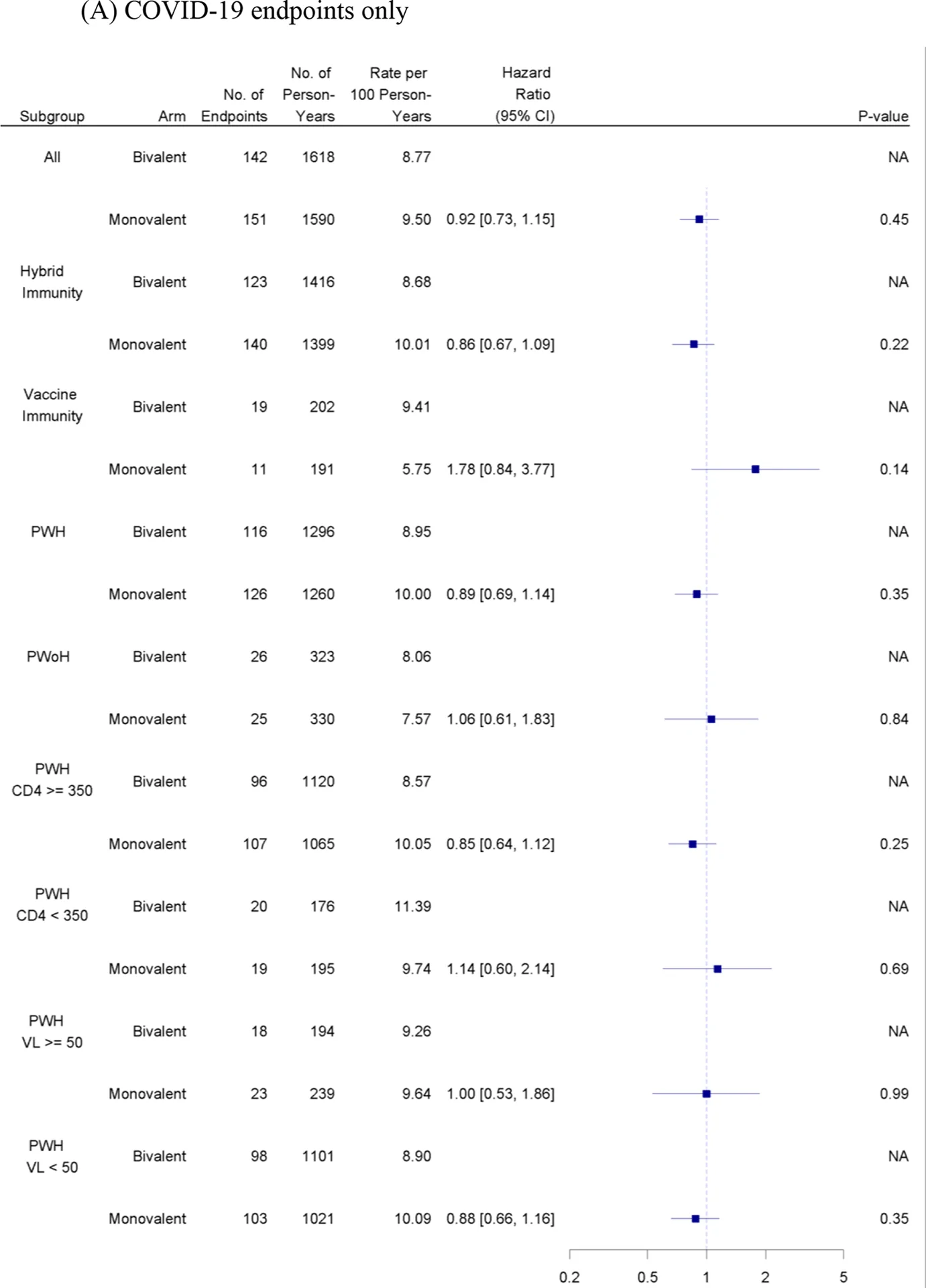

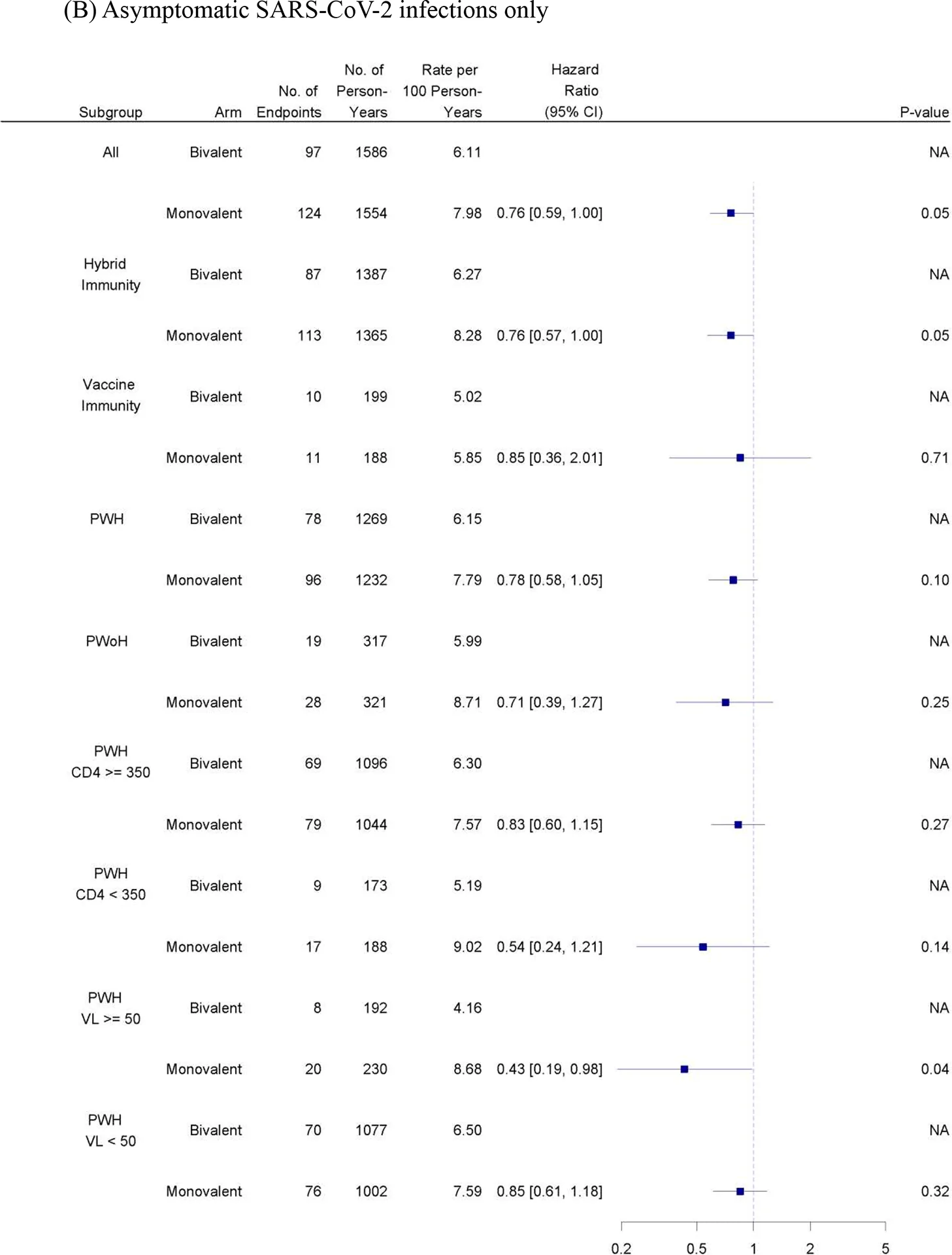

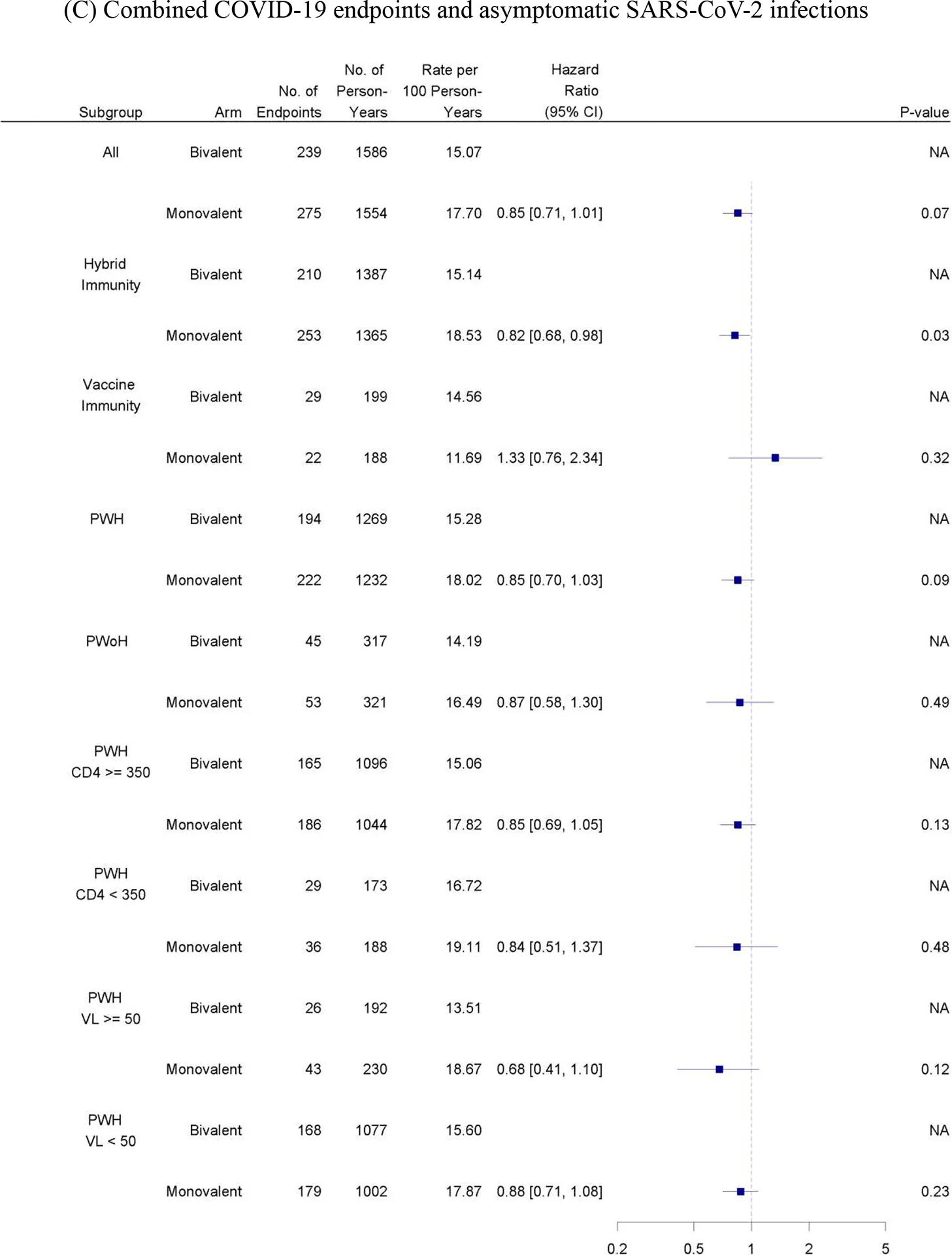
Forest plots for relative efficacy of bivalent versus monovalent booster for SARSCoV-2 incidence within 12 months after boosting

**Figure 3A-C.**
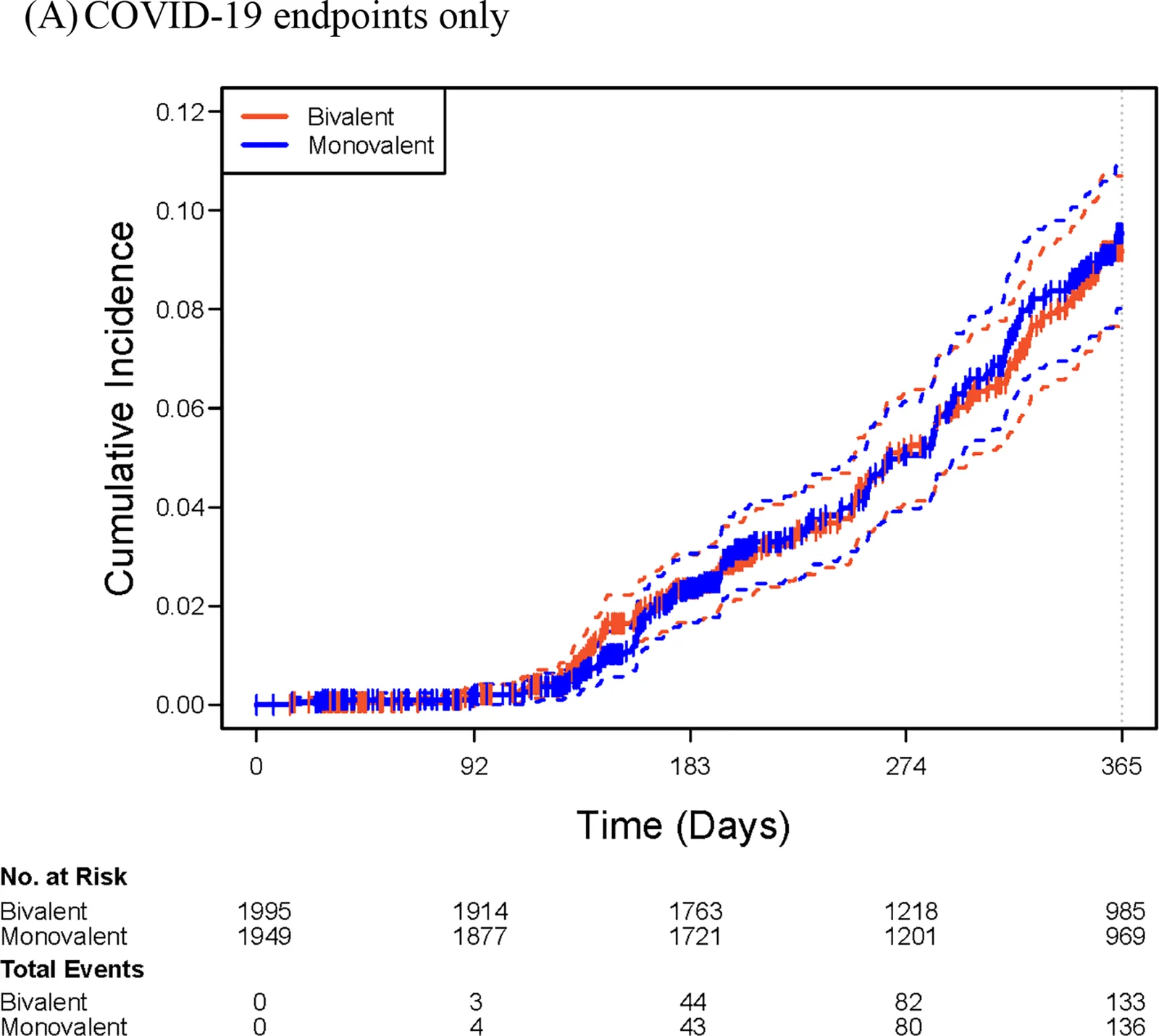

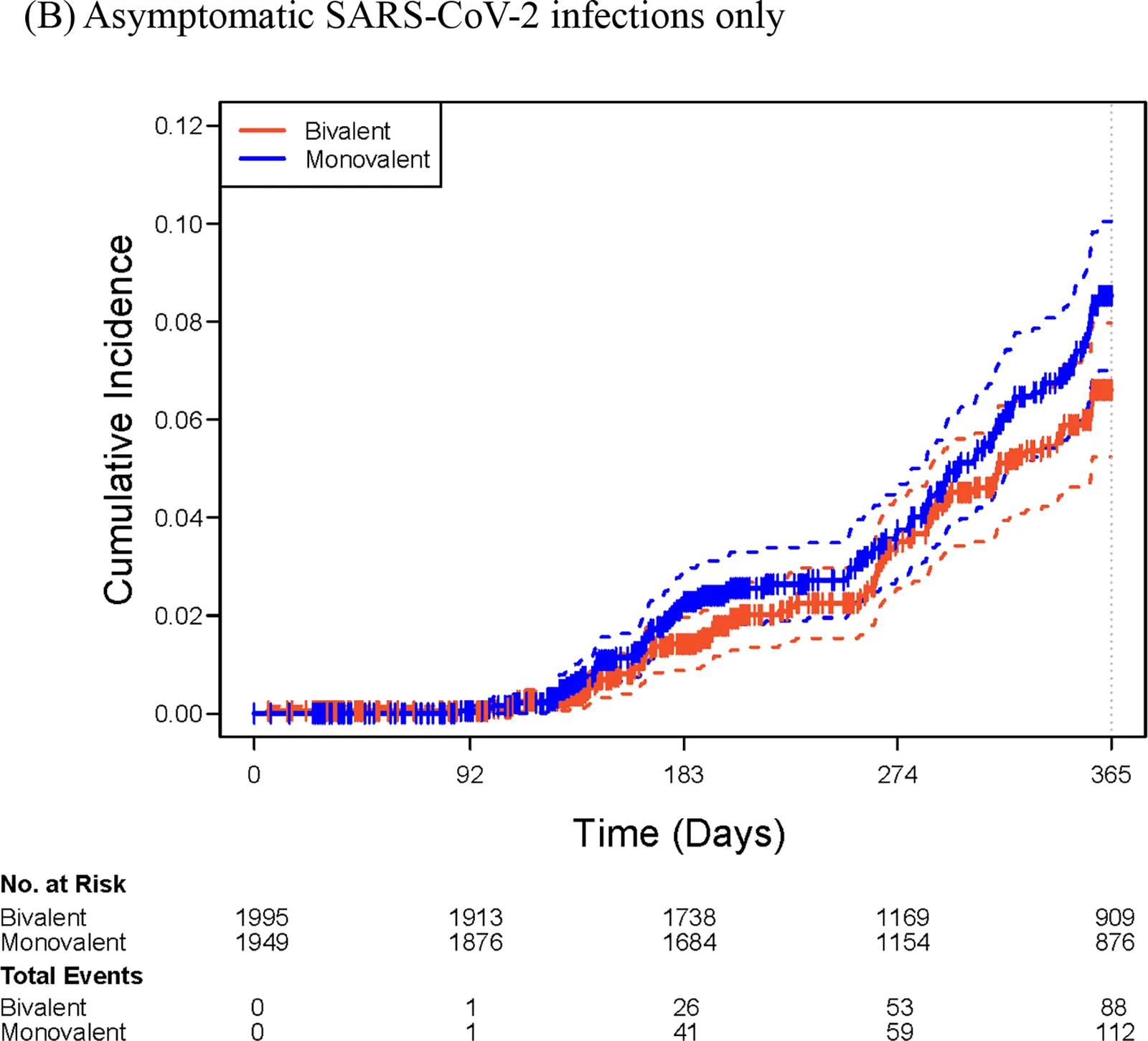

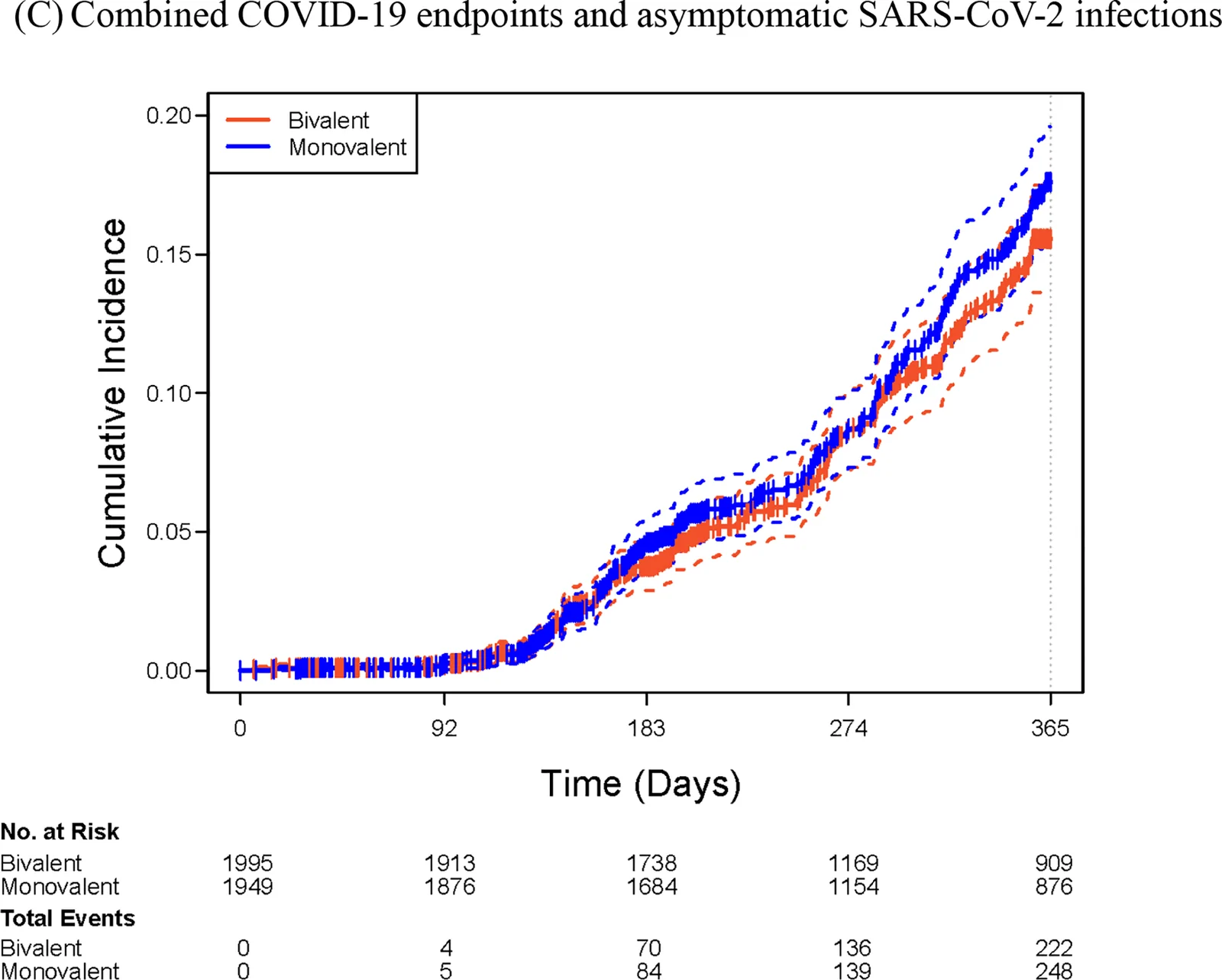
Covariate-adjusted cumulative incidence of symptomatic COVID-19 and asymptomatic SARS-CoV-2 infection within 12 months after boosting Note: Time 0 on the x-axis is Day 13 post-booster. Dashed lines indicate 95% confidence intervals.

Post-randomization and booster vaccination, 293 cases of COVID-19 were observed: 142 cases in the bivalent arm (8.77 per 100 person-years) and 151 in the monovalent arm (9.50 per 100 person-years, Fig. 2A). Only one recipient – a female, age 48 years, without HIV at baseline, in the monovalent arm – developed severe COVID-19 at month 4 but recovered without hospitalization or specific treatment. She had two oximetry readings <90%, 10 readings from 90–95%, and 11 readings >95%. The cumulative incidence at month 12 was 7.47% (95% CI 6.24–8.70) in the bivalent arm and 7.84% (6.57–9.11) in the monovalent arm (Fig. 3A). We observed no difference in the rate of COVID-19 between the bivalent and monovalent arms (HR 0.92; 95% CI 0.73–1.15; p=0.45), and no difference between arms among participants with hybrid immunity; vaccine immunity; PWH; PWoH; PWH with CD4 count ≥350 cells/mm3 or <350 cells/mm3; PWH with VL ≥50 or < 50 copies/ml (Fig. 2A). Analyses for symptomatic endpoints were repeated in the randomized cohort at month 6 (Fig. S3, S4) and in the FPP cohort at month 12 (Fig. S5, S6), with similar findings. The overall HR point estimate of 0.92 for COVID-19 was constant throughout the study, with the 95% CI always inclusive of 1.00 (Fig. 5).

**Figure 4.**
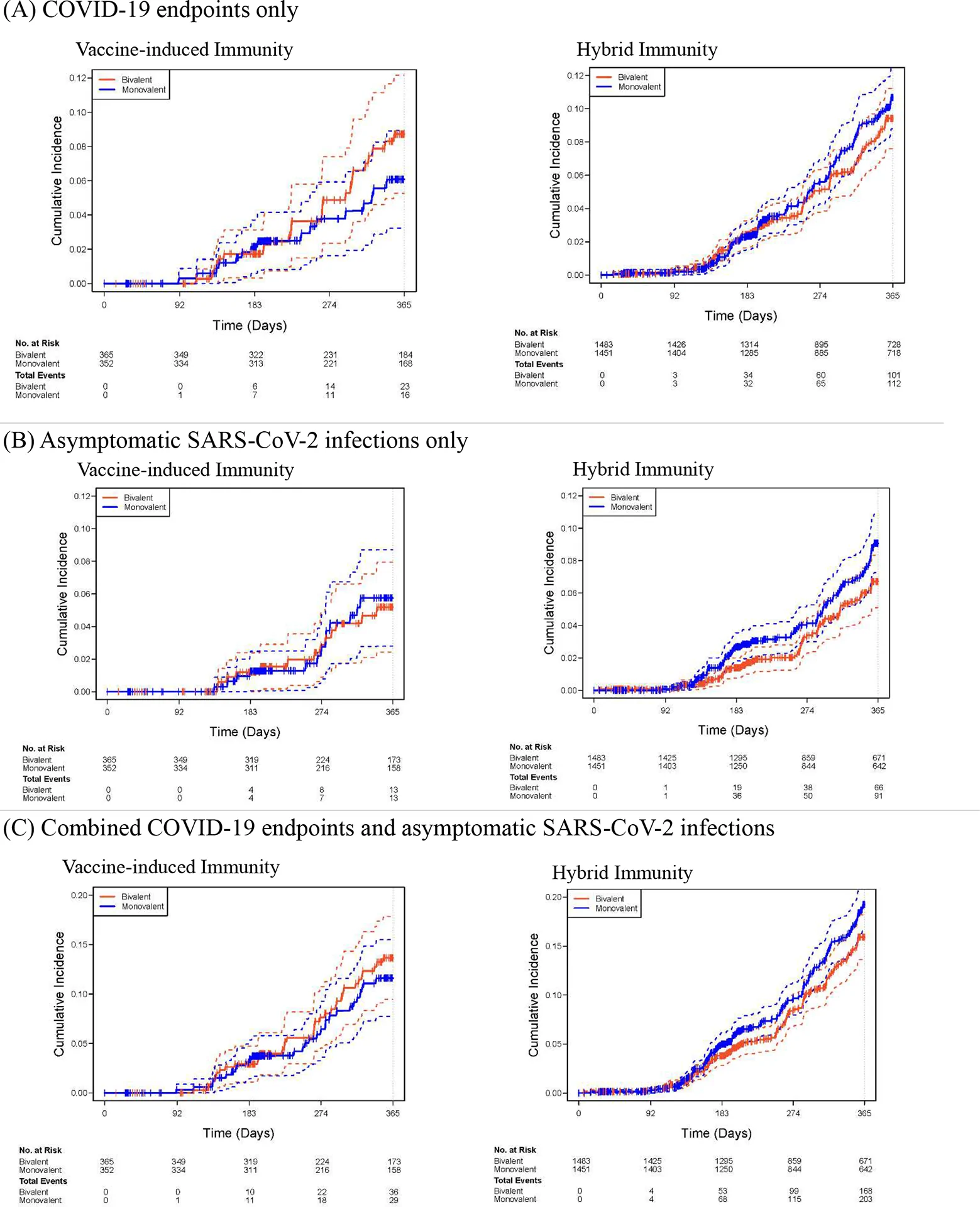
Covariate-adjusted cumulative incidence of symptomatic COVID-19 and asymptomatic SARS-CoV-2 infection after boosting among those with hybrid immunity or vaccine-induced immunity

**Figure 5.**
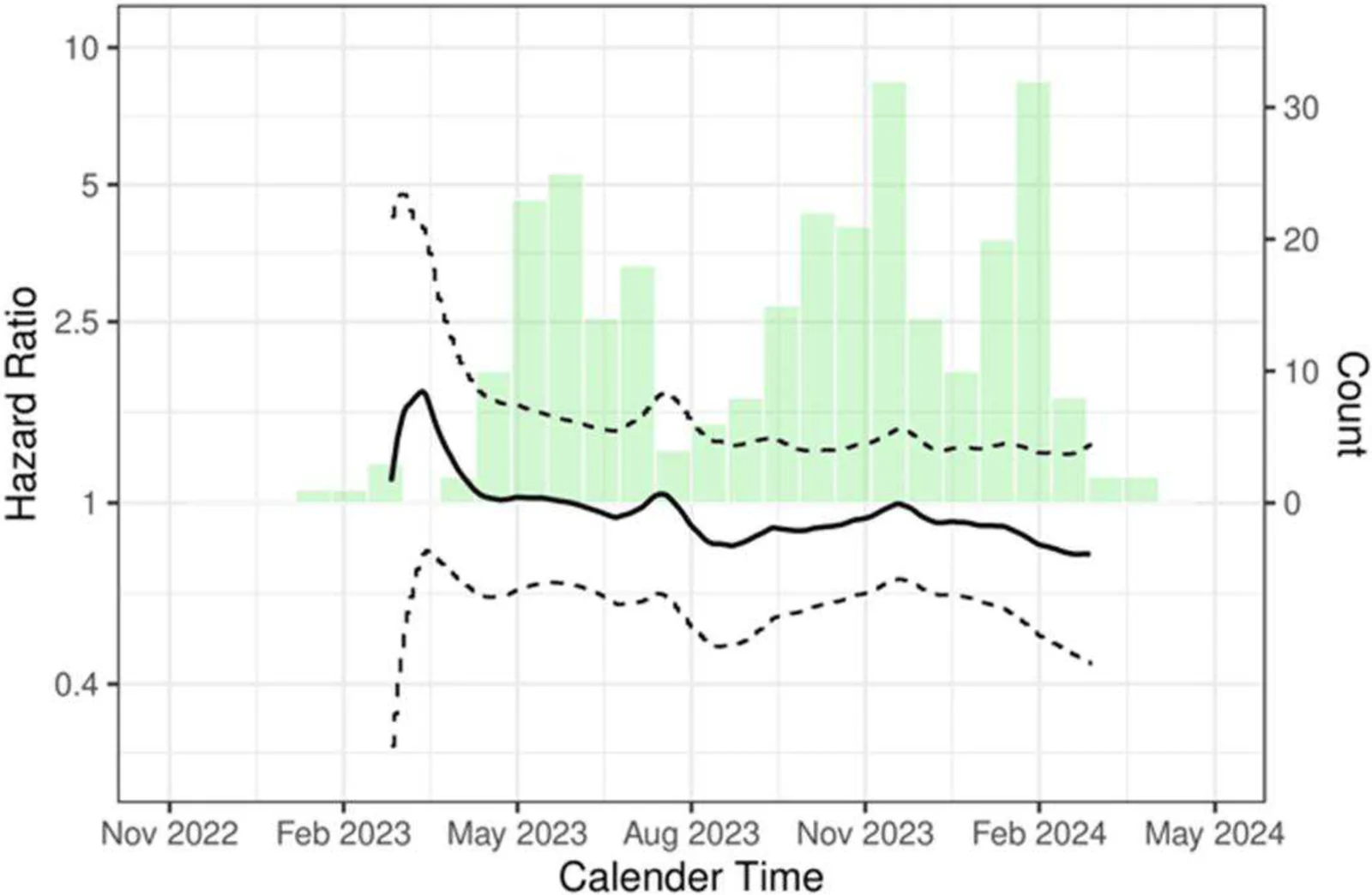
Relative vaccine efficacy of bivalent versus monovalent booster for COVID-19 over calendar time Note: Dashed lines indicate 95% confidence intervals of the relative efficacy (hazard ratio bivalent / monovalent) of the vaccines. Count (bar graph) represents cases of COVID-19 in the cohort in both arms (does not include asymptomatic infections).

In addition to the 293 cases of symptomatic COVID-19, 221 asymptomatic infections were observed: 97 in the bivalent arm and 124 in the monovalent arm (Fig. 2B, 3B). This yielded a 24% lower hazard of asymptomatic infection in the bivalent arm (HR 0.76, 95% CI 0.59–1.00, P=0.05). The benefit of reduced asymptomatic infections in the bivalent arm was significant in participants with hybrid immunity (HR 0.76, 95% CI 0.57–1.00, P=0.05) and PWH with VL ≥50 copies/mL (HR 0.43, 95% CI 0.19–0.98, P=0.04; Fig 2B, 4B). Combining symptomatic and asymptomatic SARS-CoV-2 (restricting to the first event) yielded 514 events: 239 in the bivalent arm and 275 in the monovalent arm, with a HR of 0.85 (95% CI 0.71–1.01, P=0.07). The total infection rate was significantly lower in participants with hybrid immunity (HR 0.82, 95% CI 0.68–0.98, P=0.03; Fig. 2C, 4C).

During the trial, 43 sites collected 54,185 nasal swabs, of which 905 swabs (from 668 participants) tested positive for SARS-CoV-2. Among participants with a PCR-positive swab, 8943 resting pulse oximeter readings were recorded. Among 650 participants with pulse oximeter readings (522 PWH and 128 PWoH), 65 (10.0%) experienced a reading <93%, 50 (9.6%) among PWH and 15 (11.7%) among PWoH. Of the 50 PWH, 6 had a CD4+ count of <200 cells/mm3 at baseline. Stratifying the 650 participants by study arm, 337 received monovalent boost and 313 received bivalent boost, of whom 32 (9.5%) and 33 (10.5%) experienced a pulse oximeter reading of <93%, respectively.

Of the 3,988 participants, 173 (4.3%), 105 PWH and 68 PWoH, were in the safety subset, 89 in the bivalent and 84 in the monovalent arm (Table S1). Safety and tolerability were similar between arms with 16.9% of participants in the bivalent arm and 26.2% in the monovalent arm reporting AEs. The most common reactions were pain or tenderness at the injection site (11.2% and 14.3%), headache (13.5% and 19.0%) and malaise or fatigue (11.2% and 11.9%), in the bivalent and monovalent arms, respectively. All events were mild or moderate, except for one grade 3 case of fever in the monovalent arm. There were no potentially life-threatening or fatal events attributed to the vaccines, and no statistically significant differences in solicited events by study arm. A higher proportion of PWoH reported pain or tenderness compared to PWH (20.6% vs. 7.6%, p=0.012), malaise/fatigue (17.6% vs. 7.6%, p=0.04) and myalgia (13.2% vs. 4.8%, p=0.049). Between days 0 and 28 post-baseline, two participants (2.2%) in the bivalent arm reported five events total; in the monovalent arm, two participants (2.4%) reported two events total (Table S2). Of the seven AEs, six were moderate (grade 2), none related to study vaccine; and one was vaccine-related severe injection site pain in the bivalent arm (Table S3). Events are further reported in Table S4.

Among the 3,988 participants, SAEs were experienced by 2.2% (45) of participants in the bivalent arm and 2.5% (50) in the monovalent arm (Table S4). Of the 100 SAEs among these 95 participants, none were deemed vaccine-related.

We observed 177 pregnancy outcomes among 2,709 female participants (86.4% PWH) by month 12 of the RCT: 80 in the monovalent arm and 97 in the bivalent arm (Table S5). Of these, 134 (75.7%) were full-term live births. There were 35 adverse pregnancy outcomes (13 premature live births, 16 spontaneous abortions/miscarriages [<20 weeks], 6 stillbirths [≥20 weeks]), and 7 elective abortions. There were no differences in outcomes by vaccine arm (Chi-squared P=0.48), and rates were comparable to the general population in this region^14^.

**Figure 6.**
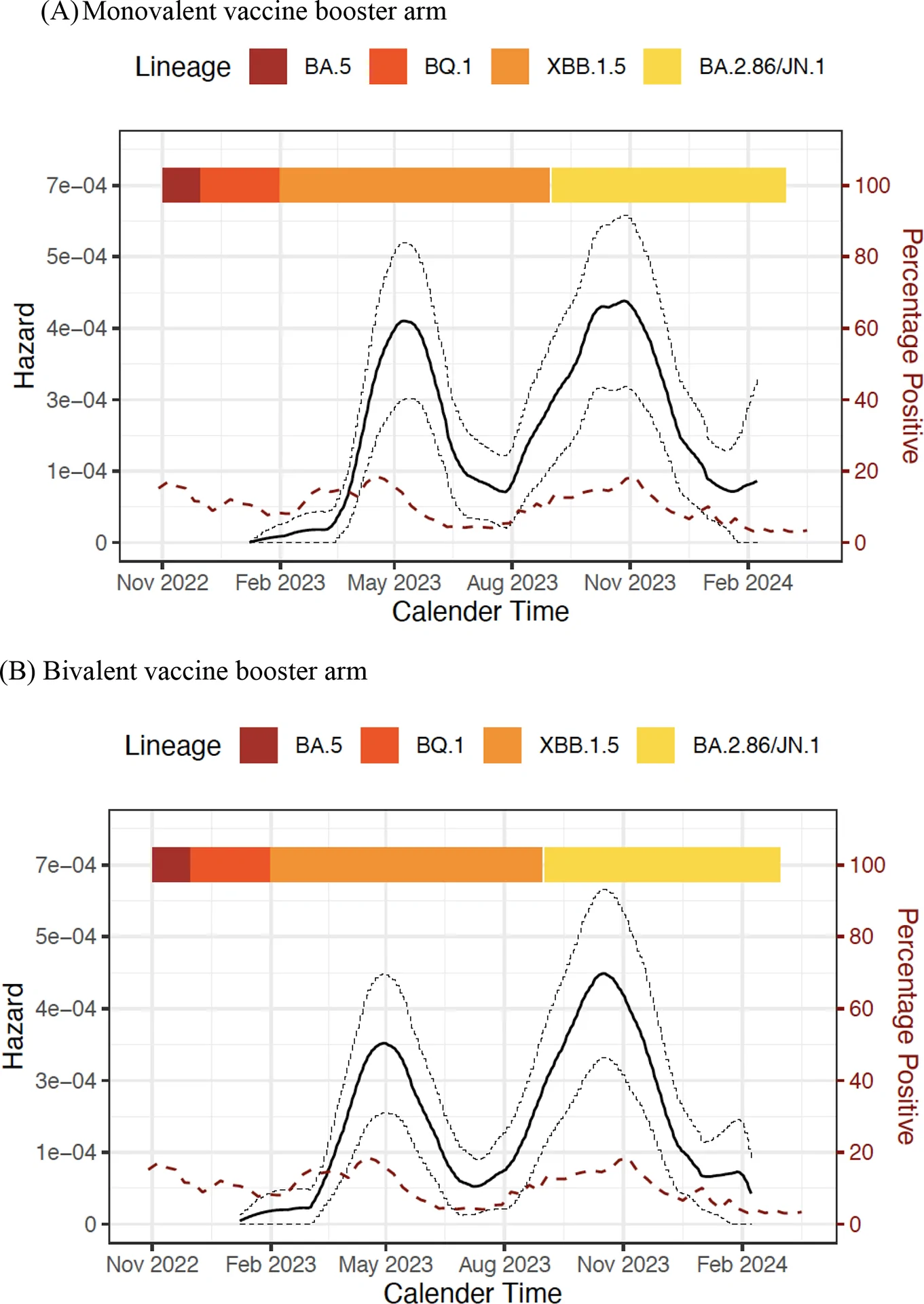

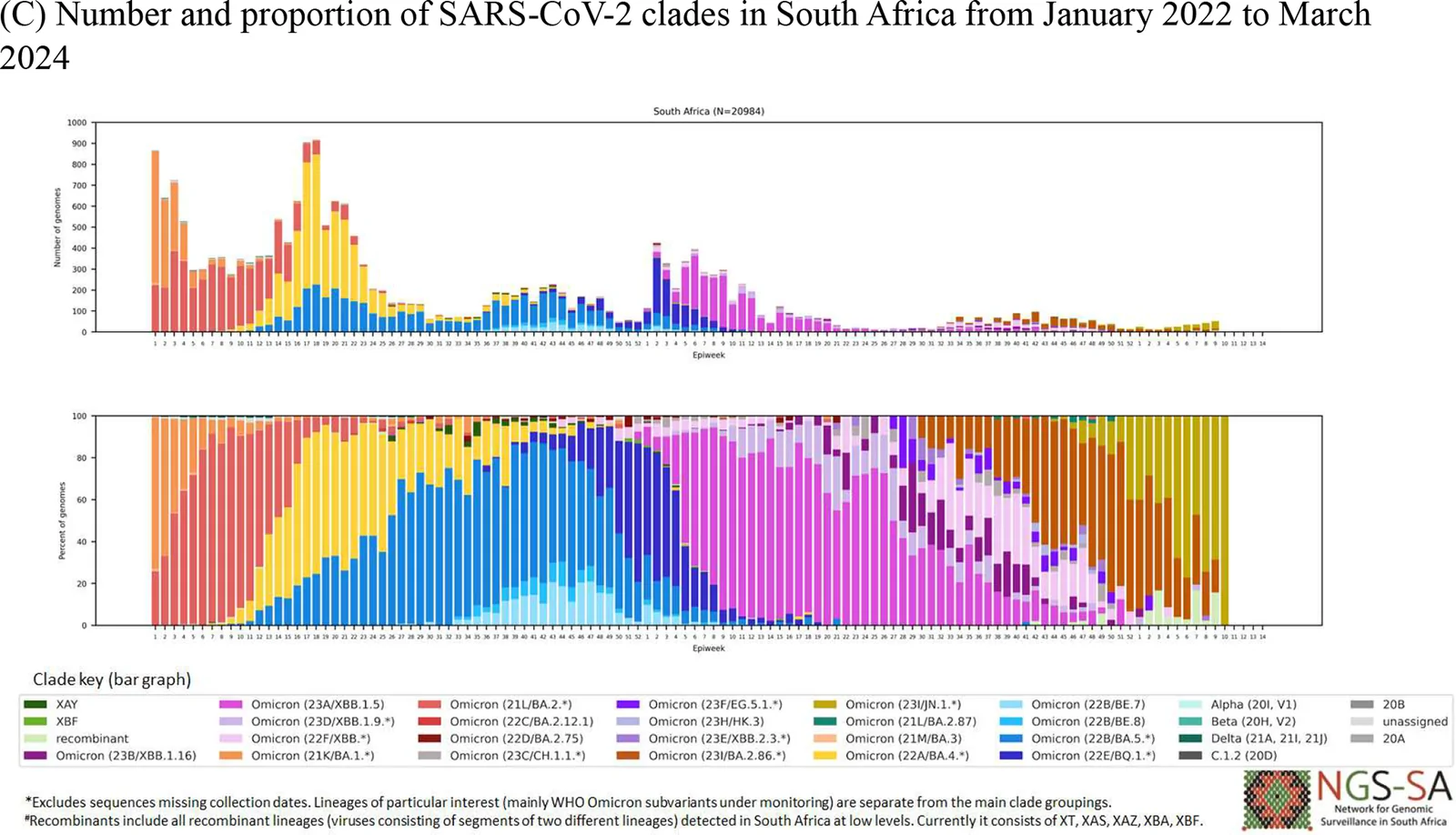
Incidence of COVID-19 by study arm over calendar time in South Africa showing nationally predominant circulating strains and percent SARS-CoV-2 test positivity among respiratory specimens Notes: Source of graphic is GISAID through the NGA-SA. Black dashed lines in 6A and 6B represent the 95% confidence intervals for the hazard of COVID-19. The red dashed line represents percent SARS-CoV-2 test positivity among respiratory specimens submitted across all South Africa Lancet Laboratories. “Lineage” in 6A and 6B refers to the one most dominant variant in South Africa nationally during that period of time, according to the GISAID data in figure 6C. 6A and 6B do not include asymptomatic infections from the trial cohort, only COVID-19 endpoints.

In South Africa, genomic surveillance data in GISAID indicated that the dominant circulating variants were BA.5 and its descendent lineage BQ.1 during the first three months, XBB.1.5 (a recombinant of two BA.2 descendent lineages) during the middle seven months, and BA.2.86 and its descendent lineage JN.1 in the latter part of the study (Fig 6C). The instantaneous risks of symptomatic COVID-19 fluctuated with introduction of a new variant and were similar between arms throughout the trial: both were low for the initial few months, then increased, mirroring the two peaks in COVID-19 positivity rates reported by Lancet Laboratories.

Characteristics of PWH in the immunogenicity subset for the nAb assessment mirrored those of the randomized cohort (Table S6). At baseline, prior to boost, nAb titers were similar between arms (Fig. 7A–B). ID50 GMTs to BA.4/5 increased from 773 to 3,639 in the bivalent arm and from 835 to 2,544 by month 1 in the monovalent arm. This represented a 4.7-fold increase (95% CI 3.8, 5.9) versus a 3.0-fold increase (95% CI 2.4, 3.8) which was 1.4-fold higher in the bivalent arm (p=0.026). ID50 GMTs to XBB.1.5 increased from 156 to 1,191 in the bivalent arm and from 135 to 559 in the monovalent arm at month 1, representing a 7.5-fold (95% CI 5.5, 10.3) versus 4.0-fold (95% CI 3.1, 5.2) increase which was 2.1-fold higher in the bivalent arm (p=0.002). The absolute titers to BA.4/5 were higher at month 1 in both arms. Restricted to participants with hybrid immunity at baseline (n=149), GMTs to BA.4/5 increased from 970 to 4,520 in the bivalent arm and from 750 to 2,625 in the monovalent arm, with the month 1 titer in the bivalent arm 1.7-fold higher (p=0.015); the corresponding values for XBB.1.5 were 194 to 1522 in the bivalent arm and 125 to 542 in the monovalent arm, with the month 1 titer 2.8-fold higher in the bivalent arm (p<0.001, Fig. 7C–D). Among those with vaccine immunity (n=51) there were no statistically significant increases in GMTs in the study arms to either BA.4/5 or XBB.1.5 strains from baseline to month 1. Analyses stratified by CD4 count (<350 and >350) did not yield observable differences or trends (Fig. S7). There was moderate correlation between nAb titer to BA.4/5 and XBB.1.5 (Spearman coefficient 0.43, Fig. S8). ID80 titers were also assessed, with similar patterns.

## DISCUSSION

This RCT, conducted during omicron waves, demonstrated the safety and utility of COVID-19 mRNA vaccine boosters in an African cohort of predominantly PWH. Incident COVID-19 was similar between the bivalent (WA1 plus BA4.5) booster and the monovalent (WA1) booster, despite a 2-fold higher increase in nAb titers to variant strains in the bivalent arm. However, a lower hazard of asymptomatic infections was observed in the bivalent arm, most apparent among participants with hybrid immunity (24% relative efficacy), resulting in a modest 15% reduction in total SARS-CoV-2 infections when symptomatic cases and asymptomatic infections were combined (18% among participants with hybrid immunity). This finding was consistent in PWH and PWoH, and generally supported results from prior comparative effectiveness research^15,16^. The superior efficacy of the bivalent vaccine against asymptomatic infections may be of public health benefit since it could curb the efficiency of SARS-CoV-2 transmission in a community or household. Both vaccines were safe and well-tolerated in the trial population.

In the 200-person subset of PWH assayed for nAbs to BA.4/5 and XBB.1.5, recipients of the bivalent booster exhibited greater increases in neutralizing titers to circulating variants than recipients of the monovalent booster, consistent with a UK-based study^17^ and COVAIL^18^. This phenomenon was especially pronounced among participants with hybrid immunity, consistent with prior immunogenicity studies (though not the UK-based study) showing that recipients of omicron-directed bivalent mRNA vaccines elicit broader and higher nAb responses, particularly if they had prior infection^19,20^. Moreover, because humoral responses are thought to play a central role in preventing SARS-CoV-2 infection while CD4+ and CD8+ T cells contribute to limiting disease severity, the greater increase in nAb titers among bivalent booster recipients may offer a mechanistic explanation for the reduction in asymptomatic infections but not COVID-19^21^. We also found a moderate correlation between anti-BA.4/5 and anti-XBB.1.5 nAb production. Further nAb testing and monoclonal antibody isolation could clarify whether the individuals who produced high titers to both sub-lineages are the same, whether exposure to bivalent vaccines promotes more nAbs to each variant, or if increased levels of somatic hypermutation produce nAbs with greater cross-reactivity.

During the initial four months after the booster dose, very low rates of COVID-19 were observed despite a coincident ~10% SARS-CoV-2 positivity in South African respiratory specimens, suggesting short-term, high efficacy of boosting with either mRNA vaccine. This is consistent with the four-month vaccine effectiveness, and subsequent waning, among bivalent mRNA vaccine recipients in a test-negative casecontrol study in California during an omicron-dominated period^23^. In the next 8 months, our study cohort’s incidence rose and fell in parallel with the background incidence, with similar risks between the two study arms and within subgroups, and no additional cases of severe COVID-19 after month 4.

These findings indicate the complexity in defining overall COVID-19 protection when strain variation, vaccination and pre-existing immunity interact. Many participants, 87.5% of whom had hybrid immunity at RCT baseline, had likely experienced multiple SARS-CoV-2 infections during the pandemic, in addition to the initial monovalent vaccine series during CoVPN 3008 Part A, and thus had well-primed immune systems. A causal inference analysis of naturally acquired immunity among residents of South African households between 2020 and 2022 estimated that nAbs mediated only 11% of protection against the omicron wave but 37% against delta, suggesting that natural immune protection against SARS-CoV-2 was not fully explained by nAbs^23^. Similarly, in a study in rural Kenya, most individuals that were seronegative for SARS-CoV-2 with no reported COVID-19 symptoms demonstrated broad T-cell reactivity to multiple SARS-CoV-2 antigens, indicating a robust cellular immune response at baseline even in the absence of detectable antibodies^24^.

The similar cumulative incidence of COVID-19 between the bivalent and monovalent booster arms was foreshadowed in the smaller COVAIL phase 2 trial conducted during circulation of BA.2-BA.5 variants^25^. Its authors found no difference in COVID-19 incidence between recipients of a prototype monovalent and an omicron-directed bivalent Moderna mRNA vaccine, hypothesizing a variety of reasons including Moderna’s high-dose formulations that elicited strong humoral and cellular immunity. Additionally, the Ubuntu trial cohort consisted of 68% women, who generally experience a reduced risk of severe COVID-19 compared to men^26^, thus potentially limiting chances of identifying a significant difference in the relative efficacy between vaccines if one exists. Finally, observational studies of booster mRNA vaccines during the omicron era have described higher vaccine effectiveness against critical illness, hospitalization, or death than against symptomatic COVID-19^27^. Given that severe disease was rare in this trial cohort, the relative efficacy endpoint relied on symptomatic COVID-19, which favored the null hypothesis.

CoVPN 3008 is the largest RCT of an mRNA COVID-19 vaccine in PWH and illustrates several important features in the mature stage of the pandemic. First is the safety of mRNA vaccines in this population. PWH reported fewer side effects than PWoH; a higher proportion of those without HIV reported injection-associated pain, malaise and myalgias, which may reflect differences in innate immune responses or perhaps more experience with local side effects from prior medical interventions. Prior studies have generally not shown a difference in reactogenicity between PWH and PWoH, although those studies had smaller cohorts^28^.

Despite close monitoring, only one severe case of COVID-19 was observed during follow-up. The trial used monthly nasal swabs and subsequently routine pulse oximetry on persons with COVID-19 to detect potential early respiratory insufficiency. Only 5% of participants with infections recorded an oxygen saturation of below 90%, suggesting that infections in this previously primed study population, during the omicron era, were not severe, with limited evidence of hypoxia, even among those with low CD4 counts.

A key strength of the trial was the direct comparison of two mRNA booster vaccines under randomized, masked conditions, thus reducing bias in outcome assessments. The trial’s location in Africa provides a valuable contribution to the literature given the relative paucity of mRNA vaccine research on the continent. We enrolled predominantly PWH from seven countries, including pregnant women and those with low CD4 counts and HIV viremia, representing a more diverse population than other mRNA vaccine trials. CoVPN 3008 was one of the largest studies investigating COVID-19 mRNA vaccines after omicron’s emergence, with 14,002 enrollees into the initial cohort study and nearly 4,000 in the RCT. Complementing relative efficacy findings with nAb data in a subset of PWH provided an important lens for comparing immunogenicity of the two boosters.

The study had some limitations. A placebo arm would have been unethical, and therefore the study could not assess the efficacy of mRNA vaccines compared to an unvaccinated population or determine if the lack of observed severe disease reflects vaccine protection, generally mild symptoms in response to omicron variants, or both. Less than 6% of participants were older than 55 years, limiting generalizability to elderly persons. Additionally, the dose of vaccines administered in this trial was 100 mcg, double what was recommended for standard use. More potent mRNA vaccines with reduced dosage are now being used for boosters. Genomic surveillance and test positivity data used to approximate background incidence and prevailing strains were derived from South Africa and may not reflect the underlying epidemiology in all trial countries, particularly because less than 1% of SARS-CoV-2 positive samples from RCT participants were sequenced. Finally, only 200 of the 3,988 participants were included in nAb assessment, which may not be a representative sample; however, baseline characteristics of the cohort and the nAb subset were reassuringly similar.

In conclusion, despite eliciting higher neutralizing titers to circulating strains of omicron, the bivalent boost (WA1 plus BA.4/5, 100 mcg) did not provide added protection against symptomatic COVID-19 over the monovalent (WA1) boost in this African cohort, most of whom were PWH with prior SARS-CoV-2 exposure. The bivalent vaccine modestly reduced asymptomatic infections, potentially mediated by antibodies. This suggests a public health benefit of booster vaccines that match circulating omicron sublineages, to reduce viral transmission. In May 2023, the World Health Organization recommended manufacturers update formulations to target recent omicron lineages in an effort to increase the breadth of SARS-CoV-2 immunity, initially recommending monovalent XBB and subsequently JN.1 lineage vaccines. Our findings support the use of monovalent vaccines that match circulating variants.

## METHODS

### Study design and participants

The RCT was conducted in Botswana, Eswatini, Kenya, Malawi, South Africa, Uganda and Zambia, at 43 clinical research sites located at diverse primary, tertiary and community care settings. Enrolment began in October 2022 and follow-up completed in May 2024. Participants in the RCT (known as Part B) were recruited from the open-label, prospective cohort study phase of CoVPN 3008 (known as Part A), which compared COVID-19 incidence in participants with hybrid immunity versus vaccine-induced immunity and correlates of risk^29–31^. The study design, including the relationship between Part A and Part B, is provided in supplemental Figure S1. There was no patient or public involvement in the design of the trial.

In Part A, participants were aged 18 years or older, self-reported as vaccine naïve self-reported to study clinic staff as either PWH or living with other comorbidities associated with severe COVID-19 outcomes^32^; expressed willingness to be followed and remain in the catchment area for the life of the study; provided written informed consent; showed willingness to undergo tests related to HIV, and to receive counseling and referrals to minimize HIV acquisition risk or improve HIV care; demonstrates understanding of the study through the Assessment of Understanding tool; and agrees not to enroll in another interventional study until after Ubuntu is completed.

All Part A participants received 100 mcg of the monovalent vaccine (mRNA-1273), six months before enrolment into the Part B RCT. Those who tested SARS-CoV-2-seronegative on a point-of-care anti-Spike serology assay received a second monovalent dose one month after Part A enrollment. Those who tested SARS-CoV-2-seropositive were not administered a second dose. SARS-CoV-2 serology was performed at the central laboratory (Bio Analytical Research Corporation [BARC], Johannesburg, South Africa) using an anti-nucleoprotein SARS-CoV-2 serology test (anti-NP; assay details provided in Supplementary Materials). Participants from Part A who had not received a 6-month booster at the time their research site secured ethical approvals for the RCT were eligible to join the RCT, provided they gave written informed consent and demonstrated understanding of the two-arm randomized design of the trial.

The trial was registered on ClinicalTrials.gov (NCT05168813) on 23 December 2021, approved by the required national ethics and regulatory bodies, and conducted with Data Safety and Monitoring Board oversight.

### Randomization and masking

Randomization sequences were computer-generated and distributed to study sites through a web-based system, assigning individual participants to the bivalent or monovalent arm in a 1:1 ratio. Randomization was stratified by study site, HIV-1 status, and SARS-CoV-2 serostatus, and performed in blocks to balance between the two study arms within these three factors. Study staff who enrolled participants did not have access to the random allocation sequence. Unmasked site pharmacists dispensed vaccines. Study staff (including research nurses, clinicians, laboratory technicians and statisticians) and participants were masked throughout the study.

### Procedures

RCT participants received a 100-mcg dose of monovalent (mRNA-1273, WA1) or bivalent (mRNA-1273.222, WA1 plus BA.4/BA.5) vaccine in the deltoid muscle from a trained, blinded study clinician, and were observed for 15 minutes; the two prepared vaccine products were visually identical to clinicians, including labeling standards. Given that the standard dose for the bivalent vaccine was 50mcg instead of 100mcg, the protocol safety review committee observed a short pause in accrual following the first 234 enrollments to review the routinely collected reactogenicity data, and upon identifying no concerns, the trial proceeded as planned.

At baseline, samples were assessed for HIV serology, HIV viral load (VL) and CD4 lymphocyte count, and SARS-CoV-2 serology and virology by real-time reverse transcription polymerase chain reaction (PCR). Follow-up visits included symptom-directed physical exams, monthly SARS-CoV-2 nasal swabs, medication review, AE reporting reminders, and pregnancy testing if indicated. Participants self-monitored for prespecified COVID-19 symptoms, and staff conducted biweekly telephonic symptom surveillance. Symptomatic individuals received PCR testing. Participants testing positive recorded daily symptoms, temperature, oxygen saturation by pulse oximeter, and had biweekly nasal swabs until negative. Clinicians provided care referrals as needed, emphasizing HIV antiretroviral therapy (ART) initiation and adherence. Concomitant care and medications, based on self-report and participant-provided health records, were captured in study data. Study staff followed pregnant participants for pregnancy outcomes.

In a safety subset of participants randomly selected per site, adverse events (AEs) were solicited for seven days post-boost and unsolicited AEs were collected up to 28 days post-boost. Serious AEs (SAEs) and adverse events of special interest (AESI, defined by the Brighton Collaboration), including cardiac and cerebral events, were captured from all participants throughout follow-up. Events were graded using the Division of AIDS Toxicity Table for Grading the Severity of Adult and Pediatric Adverse Events^33^.

### Immunological assays

Blood samples for immunological assays were collected at baseline, and months 1, 6 and 12 of the RCT (Fig. S1). Serum samples were assessed for neutralizing antibody (nAb) responses to BA.4/5 and XBB.1.5, the dominant, circulating strains in South Africa during follow-up, the country with the most study sites and most complete sequencing of isolates in the GISAID database. A subset of 100 PWH per arm from the multi-country RCT cohort was randomly selected for testing at baseline and month 1 post-booster. nAb responses were tested using a validated vesicular stomatitis virus-based pseudovirus neutralization assay that is not subject to ART interference^34^. nAb titers were calculated as the reciprocal serum dilution corresponding to the 50% inhibitory dose (ID50) and 80% (ID80) levels. Titer response frequencies [>limit of detection (LOD) = 10] and geometric mean titers (GMTs) with 95% confidence intervals (CIs) were assessed for each booster arm, and the fold-changes were calculated between baseline and month 1. A Wilcoxon rank sum test was used to determine whether titers differed between arms. A Spearman rank correlation coefficient was calculated to estimate the level of correlation of nAb titers to BA.4/5 and XBB.1.5.

### Outcomes

Efficacy endpoints were categorized according to one of three definitions: (1) the U.S. CDC COVID-19 case definition: one PCR-positive nasal swab within 14 days of the onset of at least one systemic symptom (fever ≥38°C, chills, myalgia, headache, sore throat, new loss of taste or smell), or at least one respiratory sign or symptom (cough, shortness of breath or difficulty breathing), or clinical or radiographical evidence of pneumonia; (2) severe COVID-19: a PCR-positive nasal swab, at least one sign, symptom, or other evidence of severe disease (e.g., respiratory failure, shock, intensive care unit admission) and confirmation by an independent adjudication committee; and (3) asymptomatic infections (a protocol specified secondary endpoint): a PCR-positive nasal swab at least 14 days after vaccine booster who did not meet the CDC definition for COVID-19 up until they tested negative. The date of diagnosis of lab-confirmed symptomatic COVID-19 and severe COVID-19 was the earlier date of either the first reported qualifying symptoms, or positive SARS-CoV-2 sample. The date of asymptomatic infection was the date of the first positive SARS-CoV-2 sample. Analyses included endpoints occurring from day 14 after the booster dose until the end of 12-month follow-up.

### Statistical analysis

We projected that approximately 4,200 participants would be eligible and receive the bivalent or monovalent boost, yielding ~90% power to detect a relative risk of 2.5–3.0 between arms.

Analyses of the efficacy endpoint focused on the randomized cohort and were repeated in the full per-protocol (FPP) cohort (see Supplementary Materials). The relative efficacy of the bivalent versus monovalent boosts was assessed at 6 and 12 months after the boost using a calendar-time-based semiparametric Cox proportional hazards method and a study-time-based covariate-adjusted, cumulative incidence time method, in terms of one minus the hazard ratio (HR) and one minus the marginalized relative cumulative incidence rates, respectively. Secondary analyses compared the relative vaccine efficacy among PWH, people without HIV (PWoH), persons with vaccine-induced immunity, persons with hybrid immunity, and among PWH, those with baseline CD4 counts above and below 350 cells/mm3, and those with baseline HIV VL above and below 50 copies/ml. Vaccine-induced immunity was defined as having no evidence of natural infection based on PCR, anti-NP or anti-Spike serology, and hybrid immunity was defined as having evidence of infection based on positivity on any of these tests at RCT baseline. Cox HRs were adjusted for sex, history of tuberculosis (TB) at RCT baseline and SARS-CoV-2 status at RCT baseline in the overall analysis; for analyses of PWH, CD4 count below or above 500 cells/mm3 and VL (above and below 50 copies/ml) were considered. The marginalized relative cumulative incidence rates were adjusted for recent infection within the last 6 months (i.e., a new infection diagnosed during Part A of the study), and group defined by cross-classification of HIV status at baseline and history of SARS-CoV-2 infection at RCT baseline.

To understand the context of background incidence and circulating variants in South Africa, we obtained test positivity rates from Lancet Laboratories (a major laboratory with nationally representative data), and data from the GISAID Data Science Initiative (Fig. 6C), respectively^35,36^. These analyses were limited to South Africa because it was the only African country to report national case rates for the entire RCT period, it had the greatest number of samples submitted to GISAID, and most trial sites were located there. We plotted instantaneous risk of COVID-19 by study arm, and test positivity rates over calendar time.

Instantaneous hazards of study-observed infections in both study arms, their ratio, as well as GIAIDS-based COVID-19 test positivity over calendar time were plotted from October 2022, when the first booster dose was administered, to the end of follow-up in May 2024 to investigate the relative instantaneous risk of COVID-19. These hazards were estimated using a non-parametric, kernel-smoothing-based method^37^.

## Supplementary Material

Supplementary Files

This is a list of supplementary files associated with this preprint. Click to download.

- CodePartBXXXCombinedDocument.pdf
- CodePartBXXXCombinedDocument.pdf
- CodePartBXXXCombinedDocument.pdf
- CodePartBXXXCombinedDocument.pdf
- SAPUbuntuCoVPN3008RelativeEfficacy.pdf
- SAPUbuntuCoVPN3008RelativeEfficacy.pdf
- SAPUbuntuCoVPN3008RelativeEfficacy.pdf
- SAPUbuntuCoVPN3008RelativeEfficacy.pdf
- SupplementarymaterialsUbuntuCoVPN3008RelativeEfficacy.docx
- SupplementarymaterialsUbuntuCoVPN3008RelativeEfficacy.docx
- SupplementarymaterialsUbuntuCoVPN3008RelativeEfficacy.docx
- SupplementarymaterialsUbuntuCoVPN3008RelativeEfficacy.docx
- CoVPN3008v8.0Final28June23.pdf
- CoVPN3008v8.0Final28June23.pdf
- CoVPN3008v8.0Final28June23.pdf
- CoVPN3008v8.0Final28June23.pdf

## Figures and Tables

**Figure 7A-B.**
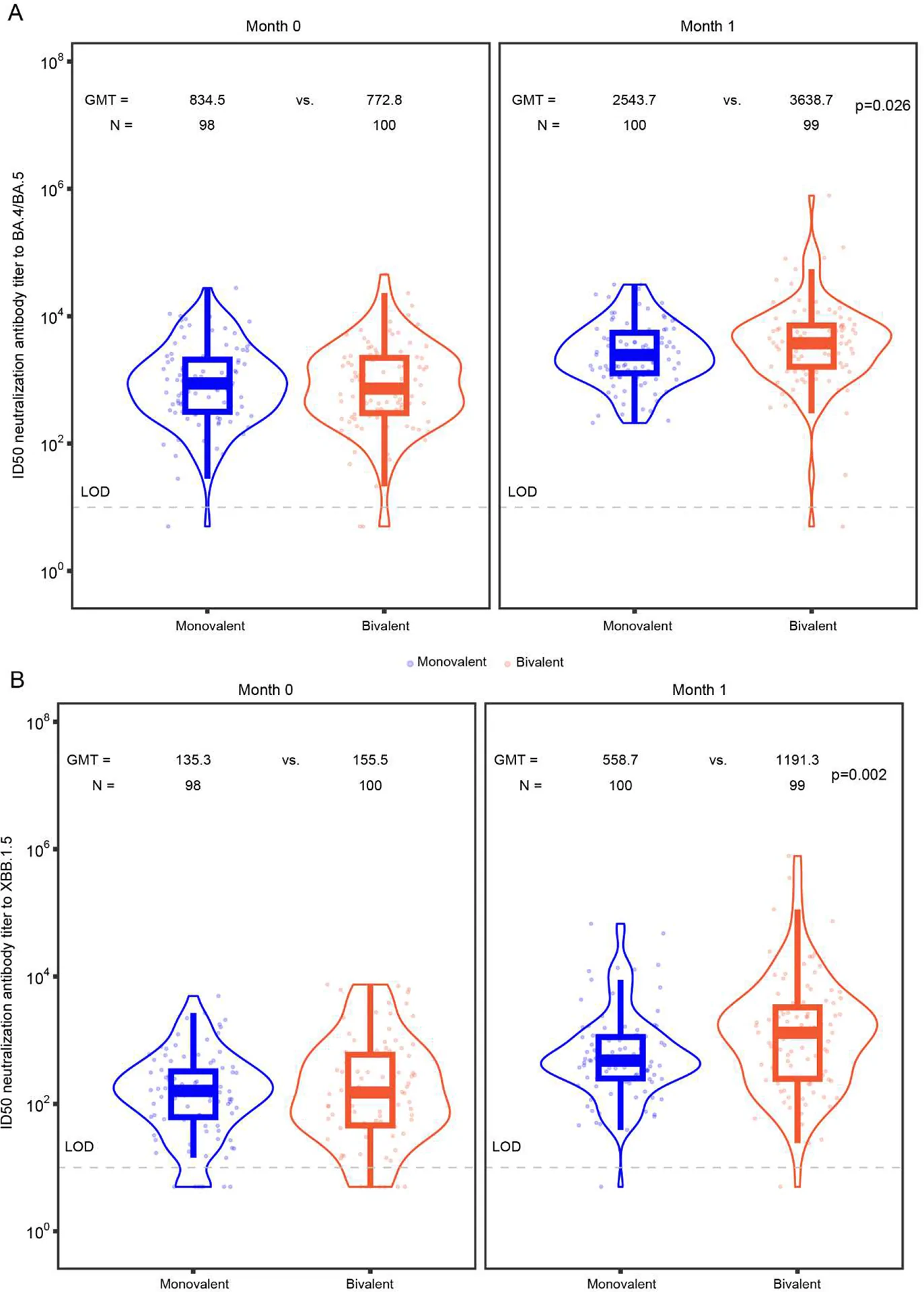
ID50 neutralizing antibody titers to BA.4/5 (panel A) and XBB.1.5 (panel B) in participants living with HIV at baseline and month 1 by study arm Note: GMT = geometric mean titer; LOD = limit of detection. P-values obtained using Wilcoxon signed rank test.

**Figure 7C-D.**
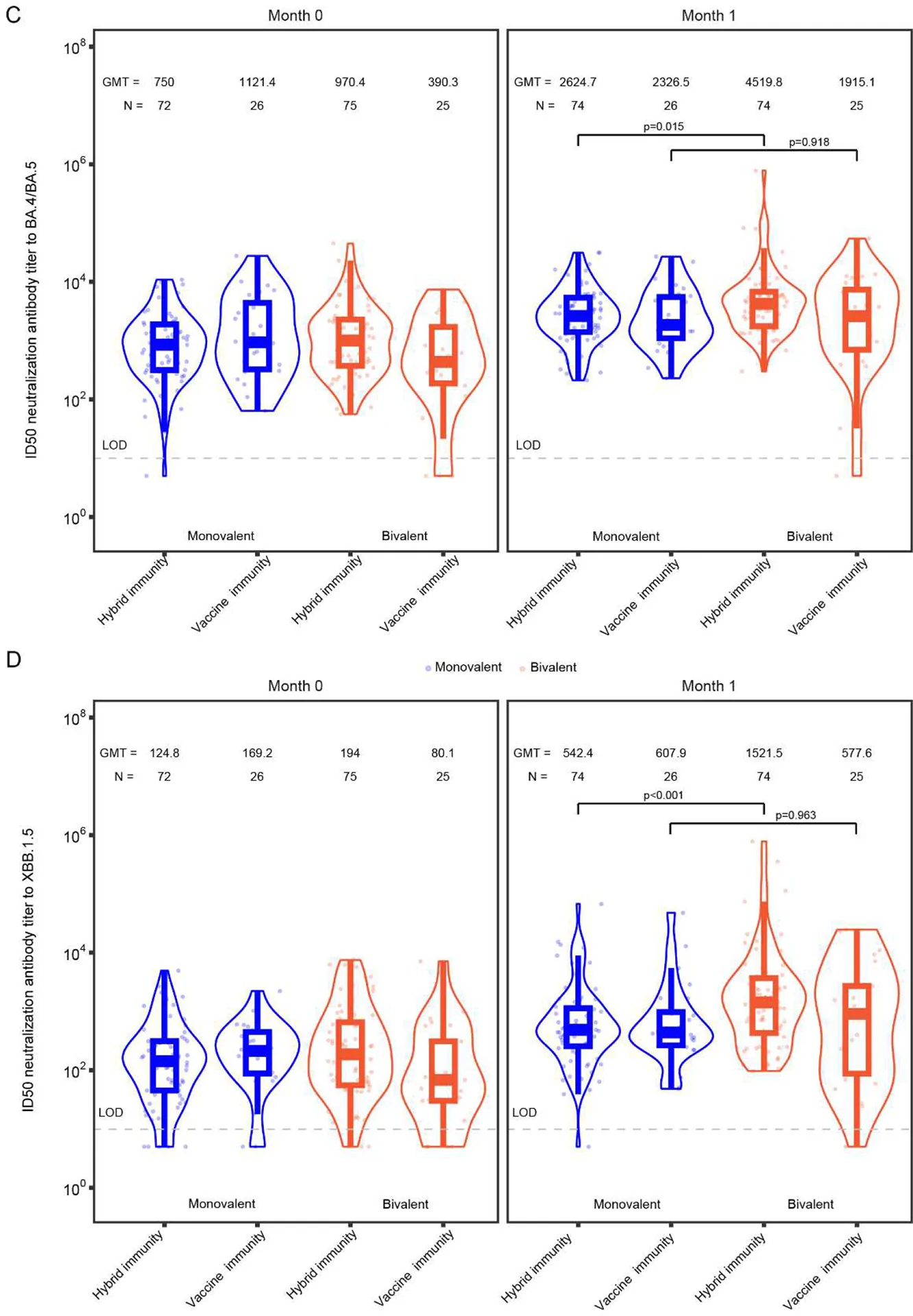
ID50 neutralizing antibody titers to BA.4/5 (panel C) and XBB.1.5 (panel D) in participants living with HIV at baseline and month 1 by study arm and baseline SARS-CoV-2 immune status (vaccine-induced immunity or hybrid immunity). Note: GMT = geometric mean titer; LOD = limit of detection. P-values obtained using Wilcoxon signed rank test.

**Table 1.** Participant demographic and clinical characteristics by vaccine arm and HIV status at trial baseline

	Total		Monovalent		Bivalent	
	Monovalent (n=1976)	Bivalent (n=2012)	PWH (n=1529)	PWoH (n=447)	PWH (n=1557)	PWoH (n=455)
**Country – n (%)**						
RSA	1431 (72.4%)	1453 (72.2%)	1029 (67.3%)	402 (89.9%)	1038 (66.7%)	415 (91.2%)
Non-RSA	545 (27.6%)	559 (27.8%)	500 (32.7%)	45 (10.1%)	519 (33.3%)	40 (8.8%)
Botswana	26 (4.8%)	31 (5.5%)	13 (2.6%)	13 (28.9%)	16 (3.1%)	15 (37.5%)
eSwatini	5 (0.9%)	6 (1.1%)	5 (1.0%)	0 (0.0%)	6 (1.2%)	0 (0.0%)
Kenya	286 (52.5%)	289 (51.7%)	273 (54.6%)	13 (28.9%)	277 (53.4%)	12 (30.0%)
Malawi	33 (6.1%)	36 (6.4%)	33 (6.6%)	0 (0.0%)	36 (6.9%)	0 (0.0%)
Uganda	180 (33.0%)	183 (32.7%)	161 (32.2%)	19 (42.2%)	170 (32.8%)	13 (32.5%)
Zambia	15 (2.8%)	14 (2.5%)	15 (3.0%)	0 (0.0%)	14 (2.7%)	0 (0.0%)
**Sex - n (%)**						
Female	1342 (67.9%)	1367 (67.9%)	1164 (76.1%)	178 (39.8%)	1176 (75.5%)	191 (42.0%)
**Age - n (%)**						
≤40 years	1217 (61.6%)	1234 (61.3%)	888 (58.1%)	329 (73.6%)	888 (57.0%)	346 (76.0%)
>40 years	759 (38.4%)	778 (38.7%)	641 (41.9%)	118 (26.4%)	669 (43.0%)	109 (24.0%)
Median (range)	37.5 (18.0, 80.0)	37.0 (18.0, 74.0)	39.0 (18.0, 69.0)	33.0 (18.0, 80.0)	39.0 (18.0, 74.0)	31.0 (19.0, 70.0)
**History of TB - n(%)**	245 (12.4%)	232 (11.5%)	221 (14.5%)	24 (5.4%)	212 (13.6%)	20 (4.4%)
CD4 count* (cells/mm ^**3**^ **) - n (%)**						
<200	95 (6.2%)	76 (4.9%)	95 (6.2%)	-	76 (4.9%)	-
200 - <350	149 (9.7%)	157 (10.1%)	149 (9.7%)	-	157 (10.1%)	-
350 - <500	263 (17.2%)	243 (15.6%)	263 (17.2%)	-	243 (15.6%)	-
≥500	1022 (66.8%)	1081 (69.4%)	1022 (66.8%)	-	1081 (69.4%)	-
Median (IQR)	656 (441, 881)	651 (450, 891)	656 (441, 881)	-	651 (450, 891)	-
HIV VL* (copies/mL) - n (%)						
<50	1223 (80.0%)	1304 (83.8%)	1223 (80.0%)	-	1304 (83.8%)	-
≥50	306 (20.0%)	250 (16.1%)	306 (20.0%)	-	250 (16.1%)	-
Missing	0 (0.0%)	3 (0.2%)	0 (0.0%)	-	3 (0.2%)	-
Median (IQR)	169 (40, 5790)	86 (40, 6011)	169 (40, 5790)	-	86 (40, 6011)	-
ART* status - n (%)						
On ART	1468 (96.0%)	1497 (96.1%)	1468 (96.0%)	-	1497 (96.1%)	-
Not on ART	61 (4.0%)	60 (3.9%)	61 (4.0%)	-	60 (3.9%)	-
**SARS-CoV-2 status - n (%)**						
Hybrid immunity (+ serology or + virology)	1732 (87.7%)	1759 (87.4%)	1333 (87.2%)	399 (89.3%)	1359 (87.3%)	400 (87.9%)
Vaccine immunity	244 (12.3%)	253 (12.6%)	196 (12.8%)	48 (10.7%)	198 (12.7%)	55 (12.1%)
Recent infection (+ virology) in the 6 months before boost	301 (15.2%)	312 (15.5%)	226 (14.8%)	75 (16.8%)	241 (15.5%)	71 (15.6%)

## Data Availability

Permission to access deidentified data, data dictionary and other relevant analysis files can be requested from the HIV Vaccine Trials Network (HVTN) and the Statistical Center for HIV/AIDS Research & Prevention (SCHARP) after publication, with a signed data access agreement. The Study Protocol (including sample consent form), the Statistical Analysis Plan (SAP) and Statistical Code are provided in the Supplemental Materials for this Article. .
